# Antimicrobial Activity and Mechanisms of Punicalagin against *Vibrio parahaemolyticus*

**DOI:** 10.3390/foods13091366

**Published:** 2024-04-28

**Authors:** Hongli Liu, Wenxiu Zhu, Yue Zou, Xiaodong Xia

**Affiliations:** State Key Laboratory of Marine Food Processing and Safety Control, National Engineering Research Center of Seafood, School of Food Science and Technology, Dalian Polytechnic University, Dalian 116034, China; lhl2015@126.com (H.L.); zwenxiu1005@163.com (W.Z.); 1717010103@xy.dlpu.edu.cn (Y.Z.)

**Keywords:** punicalagin, *Vibrio parahaemolyticus*, cell envelope, bacterial proteins

## Abstract

This study sought to explore the antimicrobial activity of punicalagin against *V. parahaemolyticus* and its potential modes of action. *V. parahaemolyticus* ATCC 17802 and RIMD 2210633^Sm^ were exposed to punicalagin, and the energy production, membrane potential, and envelope permeability, as well as the interaction with cell biomolecules, were measured using a variety of fluorescent probes combined with electrophoresis and Raman spectroscopy. Punicalagin treatment disrupted the envelope integrity and induced a decrease in intracellular ATP and pH. The uptake of 1-N-phenyl-naphtylamine (NPN) demonstrated that punicalagin weakened the outer membrane. Punicalagin damaged the cytoplasmic membrane, as indicated by the membrane depolarization and the leakage of intracellular potassium ions, proteins, and nucleic acids. Electronic microscopy observation visualized the cell damage caused by punicalagin. Further, gel electrophoresis coupled with the Raman spectrum assay revealed that punicalagin affected the protein expression of *V. parahaemolyticus*, and there was no effect on the integrity of genomic DNA. Therefore, the cell envelope and proteins of *V. parahaemolyticus* were the assailable targets of punicalagin treatment. These findings suggested that punicalagin may be promising as a natural bacteriostatic agent to control the growth of *V. parahaemolyticus*.

## 1. Introduction

*Vibrio parahaemolyticus* is a common Gram-negative pathogen. It naturally inhabits estuaries and marine and aquaculture environments and is prevailingly found in aquatic products, with the positive detection frequently being more than 50% during the warm months [1,2]. In China, *V. parahaemolyticus* is the main cause of foodborne outbreaks, accounting for 6.7% of total outbreaks and 13.3% of total illnesses during 2011–2021. The World Health Organization has reported that *V. parahaemolyticus* is the leading cause of bacterial gastroenteritis associated with the consumption of seafood products, with its outbreaks mainly related to the global spread of the serotype O3:K6 pandemic clone [3]. Therefore, there is growing concern for adopting measures to prevent food bacterial contamination and the proliferation by *V. parahaemolyticus*.

Thus far, unremitting efforts and attempts have been made to prevent the growth of pathogenic microorganisms. Admittedly, chemical disinfectants or preservatives have been among the most common strategies, playing important roles in preventing food bacterial contamination. However, consumers’ preference for natural additives and doubts about the safety of artificial preservatives have prompted the food industry to search for natural alternatives. In addition, the emergence of extensive resistant *V. parahaemolyticus* also necessitates the development of novel antibacterial strategies [4,5]. Studies of the antibacterial activity and related applications of natural extracts, especially those from plants, are increasing worldwide [6,7]. Plant extracts rich in polyphenols have been widely researched as an alternative to chemical preservatives due to their powerful antioxidant and antibacterial properties. They can inhibit the growth of pathogenic and spoilage organisms and slow down the oxidation of nutritional ingredients and the discoloration of red meat and certain fish [8,9].

Punicalagin, a natural polyphenol isolated from pomegranate peel, has been reported to have multifarious health benefits and a wide spectrum of antimicrobial properties against several fungal and bacterial pathogens [10,11,12]. Nonetheless, little information is available regarding the antibacterial mechanism of punicalagin against *V. parahaemolyticus*. Hence, this study investigated the possible mechanisms of punicalagin against *V. parahaemolyticus* by exploring the effects of punicalagin on the bacterial energy metabolism, membrane permeability, and cell morphology, as well as its interaction with cell biomolecules of *V. parahaemolyticus*.

## 2. Materials and Methods

### 2.1. Bacterial Strains and Culture Conditions

*V. parahaemolyticus* ATCC 17802, ATCC 33847, and a streptomycin-resistant RIMD 2210633 isolate (RIMD 2210633^Sm^) were grown in tryptic soy broth (TSB, Hope Bio-technology, Qingdao, China) supplemented with 3% NaCl at 37 °C. Streptomycin sulfate (200 μg/mL, Macklin, Shanghai, China) was added for *V. parahaemolyticus* RIMD 2210633^Sm^. The cultures were centrifuged (5000× *g*, 4 °C, 5 min), washed, and then adjusted OD_600 nm_ to 0.5, corresponding to a density of approximately 10^8^ CFU/mL, which was used for subsequent experiments.

### 2.2. Determination of Minimum Inhibitory Concentration (MIC) and Minimum Bactericidal Concentration (MBC)

The MIC of punicalagin against *V. parahaemolyticus* was determined via a broth microdilution method as previously reported [13]. For the MBC, equivalent volumes (200 μL) of punicalagin (CAS# 65995-63-3, Must Bio-technology, Chengdu, China) and bacterial suspension (~10^6^ CFU/mL) were added to a sterile 96-well microplate and incubated at 37 °C for 24 h. The final punicalagin concentrations were not lower than the MIC. Then, 100 μL of suspension that showed no visible bacterial growth was spread on 3% NaCl TSA plates and incubated at 37 °C for 48 h. The lowest concentration that killed the initial inoculums by 99.9% was recorded as MBC.

### 2.3. Bacterial Growth Assay

*V. parahaemolyticus* was resuspended in fresh 3% NaCl TSB containing 1×, 2×, and 4× MIC punicalagin at a final cell concentration of approximately 10^6^ CFU/mL. Cell cultures without punicalagin were used as negative control. Samples were cultured in a shaking incubator at 37 °C at 190 rpm. At defined time points of 2, 4, and 8 h, samples were gradually diluted by 10-fold with 0.85% sterile saline spread (100 μL) onto 3% NaCl TSA plates, and the colonies were calculated via the plate counting method.

### 2.4. Intracellular ATP Assay

Intracellular ATP was detected according to the method described previously with some modifications [14]. A total of 500 μL of adjusted bacterial suspensions (10^8^ CFU/mL) with 500 μL of punicalagin at final concentrations of 0 (control), 1×, 2×, and 4× MIC were mixed in phosphate-buffered saline (PBS, pH 7.2–7.4) and cultured at 37 °C for 30 min. A total of 100 μL of mixture was added to 900 μL of PBS and then centrifuged (5000× *g*, 4 °C, 5 min) to remove the supernatants. Cell sediments were added with 1 mL extracting solution, kept cold on ice, and disrupted with a sonicator (Scientz, Ningbo, China) for four cycles of 10 s of run and 5 s of pause at a power level of 200 w. Whereafter, samples were centrifuged at 10,000×g for 10 min at 4 °C, and the supernatants were taken into another EP tube. A total of 500 μL of chloroform was added and thoroughly mixed, centrifuged (10,000× *g*, 3 min, 4 °C), and then the supernatants were collected and stored at 4 °C until measurement. Intracellular ATP concentration was measured using an ATP assay kit (Solarbio, Beijing, China) according to the instructions.

### 2.5. Intracellular pH Determination

Intracellular pH of *V. parahaemolyticus* was tested using a spectrofluorometric method referenced elsewhere [15,16]. Cells were collected and washed twice with HEPES buffer (Biological Industries, Beit HaEmek, Israel). The fluorescence probe, 2′,7′-bis-(2-carboxyethyl)-5-(and-6)-carboxyfluorescein acetoxymethyl ester (BCECF AM, Beyotime, Shanghai, China), at a final concentration of 3.0 μM, was added to 10 mL of the above buffer for 30 min in the dark at 37 °C. Cells loaded with the fluorescent probe were washed twice with PBS and resuspended in punicalagin solutions configured with PBS. Cell suspension was incubated for 1 h in the dark at 37 °C and then transferred into black 96-well microplates. Fluorescence intensities were measured with a multi-mode microplate reader (Infinite 200 Pro, Tecan, Switzerland; Ex 485 nm; Em 535 nm). The results are presented as relative fluorescent units by deducting the fluorescence background value of the cell-free groups with different concentrations of punicalagin.

### 2.6. Membrane Potential Determination

*V. parahaemolyticus* cells were collected and resuspended in PBS. The 3.0 μM probe, bis-(1,3-dibutylbarbituric acid) trimethine oxonol (DiBAC_4_(3), meilunbio, Dalian, China), was added to the bacterial suspension and incubated in the dark at 37 °C for 30 min, followed by the addition of punicalagin (0, 1×, 2×, 4× MIC). At 1 h, fluorescence intensities were measured by a Tecan multi-mode microplate reader (Ex 485 nm; Em 535 nm). Background fluorescence of the cell-free groups was determined, and the results were normalized to the corresponding background.

### 2.7. Cell Contents Release Assay

Cells were collected and resuspended in sterile saline. Equivalent volumes (6 mL) of bacterial suspensions and serial two-fold dilutions of punicalagin—to give final concentrations of 0, 1×, 2×, and 4× MIC—were incubated at 37 °C for 1 h with 190 rpm shaking. For intracellular potassium efflux, the supernatants were obtained via centrifugation (8000× *g*, 10 min, 4 °C), filtered with a 0.22 μm membrane filter, and then detected by an atomic absorption spectrophotometer (Hitachi ZA3000, Tokyo, Japan). The standard curve for calculating potassium concentration in the supernatant was obtained by a set of KCl standard solutions with concentration gradients of 10.0, 8.0, 6.0, 4.0, 3.0, 2.0, 1.0, and 0 mg/L. For macromolecular leakage, 4 mL of bacterial suspensions were centrifuged for 2 min at 12,000 rpm. The supernatants were collected, and the leakage of nucleic acids were determined at 260 nm using a microvolume spectrophotometer (NanoDrop One, Thermo Fisher Scientific, Waltham, MA, USA). The concentrations of nucleic acids in the supernatant were calculated by subtracting the background absorption of sterile saline containing the same concentration of punicalagin. The concentrations of released proteins in the supernatant were quantified by a microplate reader (Infinite 200, Tecan, Männedorf, Switzerland) according to the instruction of a detergent-compatible Bradford protein assay kit (Beyotime, Shanghai, China).

### 2.8. Permeability of the Outer Membrane

The outer membrane permeability of *V. parahaemolyticus* cells was determined using an NPN uptake assay as previously described [17]. The 1 mL *V. parahaemolyticus* suspensions (~10^8^ CFU/mL) supplemented with punicalagin (0, 1×, 2×, 4× MIC) were incubated at 37 °C with 190 rpm shaking. After 2, 4, and 8 h, cell suspension was centrifugated (5000× *g*, 4 °C, 5 min), washed, and resuspended in HEPES buffer, followed by the addition of 10 μL NPN (0.5 mM in acetone). The suspension was incubated for 10 min in the dark at room temperature, and fluorescence intensities were measured (Ex 360 nm; Em 465 nm).

### 2.9. Membrane Integrity Assay

The bacterial suspension was treated with various concentrations of punicalagin (0, 1×, 2×, 4× MIC) and cultured for 2 h at 37 °C. The *V. parahaemolyticus* cells were collected via centrifugation (10,000× *g*, 4 °C, 2 min), washed, and resuspended in PBS. Afterward, cells were stained with PI (30 μM) and SYTO (10 μM) at room temperature without light for 20 min. The cell samples were washed thoroughly with PBS to remove free probes and examined using a fluorescence microscope (Revole, Echo, San Diego, CA, USA). Confocal images of green (SYTO) and red (PI) were observed with the Overlay mode.

### 2.10. Field Emission Scanning Electron Microscope Observation (FE-SEM)

Cells (~10^8^ CFU/mL) were treated with 0, 1×, 2×, and 4× MIC punicalagin and incubated for 4–6 h at 37 °C. Subsequently, the cells were collected (5000× *g*, 4 °C, 5 min) and fixed with 2.5% glutaraldehyde at 4 °C for 4–6 h. After being washed three times with sterile saline solution, the cells were dehydrated with serially increasing concentrations of ethanol (30, 50, 70, 80, 90, and 100%) for 15 min each. The cells were air dried for overnight, coated with gold, and then visualized with FE-SEM (SU8010, Hitachi, Tokyo, Japan).

### 2.11. Bacterial Protein and DNA Assay

Bacterial protein of *V. parahaemolyticus*, treated with punicalagin (0, 1×, 2×, 4× MIC) for 6 h, was extracted via a Gram-negative bacteria protein extraction kit (BestBio, Shanghai, China) and determined with a BCA protein assay kit (Beyotime, Shanghai, China). A total of 40 μL of the extracted protein was mixed with 10 μL of 5× loading buffer (Beyotime, Shanghai, China), vibrated, and then boiled for 5 min. SDS-PAGE was performed with 10 μL of protein-loading buffer supernatant on SDS-PAGE precast gels (Tris-Gly, 4–20%, Beyotime, Shanghai, China) at a constant voltage of 100 V for 100 min. The gels were then stained with Coomassie brilliant blue R250 and scanned with a ChemiDoc Touch imaging system (Bio-Rad, Shanghai, China). Genomic DNA was extracted using a SteadyPure Universal Genomic DNA Extraction kit (Accurate biology, Changsha, China). The DNA concentration and purity were measured using a NanoDrop One spectrophotometer. The DNA integrity was detected by 1% agarose gel electrophoresis (AGE) at 90 V for 25 min.

### 2.12. Raman Spectrum Assay

The LabRAM HR Evolution Raman Spectrometer (HORIBA Scientific, Paris, France) was used to investigate the changes in the biochemical compositions of *V. parahaemolyticus* cells treated with punicalagin. *V. parahaemolyticus*, grown to log phase with punicalagin, was centrifuged, washed, and resuspended in sterilized water. The bacteria suspension was individually deposited on an aluminium-coated chip. Then, the chip was mounted on the microscope stage. A DPSS laser with 100× objective (Olympus, Tokyo, Japan) was used for focusing on the samples to collect Raman information. The test parameter was set with an excitation wavelength of 532 nm and a laser intensity of 25% over a simultaneous Raman shift from 500 to 2500 cm^−1^. Raman spectral processing of raw data were carried out using the LabSpec 6 software. The polynomial background fit and baseline subtraction were processed for background fluorescence removal. The average Raman spectrum was used for analysis and plotting via Origin pro 9.0 software (Origin Lab Corp., Northampton, MA, USA).

### 2.13. Data Analysis

All experiments were performed at least in triplicate. The data were analyzed using SPSS statistics 22.0 (IBM, Armonk, NY, USA). Results are presented as mean values ± standard deviation. Differences between the two groups were evaluated via Student’s *t* test, and differences among the groups were evaluated via Tukey HSD test.

## 3. Results

### 3.1. Antibacterial Activity

The MBCs of punicalagin were 200 μg/mL, 200 μg/mL, and 300 μg/mL for *V. parahaemolyticus* ATCC 17802, ATCC 33847, and RIMD 2210633^Sm^, respectively (Table 1). *V. parahaemolyticus* ATCC 17802 and RIMD 2210633^Sm^ were used for the following study.

**Figure 1:**
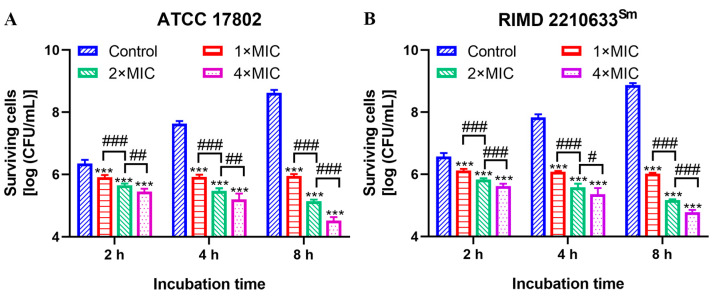
Surviving population of *V. parahaemolyticus* ATCC 17802 (**A**) and RIMD 2210633^Sm^ (**B**) during exposure to different concentrations of punicalagin. *** *p* ≤ 0.001 versus the control; ^#^ *p* < 0.05, ^##^ *p* ≤ 0.01, ^###^ *p* ≤ 0.001 for comparison between punicalagin treatments.

Punicalagin markedly restrained the growth of *V. parahaemolyticus* ATCC 17802 and RIMD 2210633^Sm^ (*p* < 0.001) in a dose- and time-dependent manner. Bacterial counts in the control continued to grow with the extension of incubation time, reaching 8.63 or 8.87 log CFU/mL for *V. parahaemolyticus* ATCC 17802 or RIMD 2210633^Sm^ at 8 h (Figure 1). However, *V. parahaemolyticus* growth virtually stopped following treatment with 1× MIC punicalagin, as evidenced by bacterial counts which were almost identical to the initial inoculation. Moreover, *V. parahaemolyticus* counts were significantly fewer (*p* < 0.01) at 2× or 4× MIC punicalagin than the initial inoculation. Compared to the control, punicalagin treatment significantly reduced the bacteria number by 1.72 to 2.47 log CFU/mL at 4 h (*p* < 0.001). After 8 h, punicalagin caused a greater decrease of 2.67, 3.49, and 4.11 log CFU/mL for the ATCC17802 strain (Figure 1A), as well as 2.85, 3.71, and 4.09 log CFU/mL for RIMD 2210633^Sm^, compared to the control (*p* < 0.001) (Figure 1B).

### 3.2. Effects of Punicalagin on Energy Metabolism

**Figure 2:**
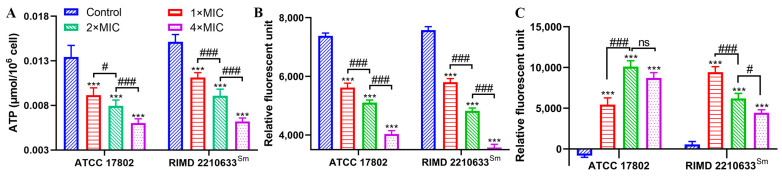
Changes in intracellular ATP (**A**), pH (**B**), and membrane potential (**C**) of *V. parahaemolyticus* ATCC 17802 and RIMD 2210633^Sm^ following punicalagin treatment. *** *p* ≤ 0.001 versus the control; ^#^ *p* < 0.05, ^###^ *p* ≤ 0.001 for comparison between punicalagin treatments. ns, no significant difference.

The intracellular ATP concentration of both the ATCC 17802 and RIMD 2210633^Sm^ strains treated with punicalagin showed a significant reduction (*p* < 0.001) of 31.1–54.6% and 26.4–59.1%, respectively, compared to the control (Figure 2A). Similar results were observed in intracellular pH. The maintenance of pH homeostasis is essential for a variety of cellular metabolic processes, including bacterial growth, signal transduction, and enzyme activity [18,19]. However, the relative fluorescent intensity of *V. parahaemolyticus* ATCC 17802 and RIMD 2210633^Sm^ dramatically declined by 23.8–45.3% and 23.4–52.8%, respectively, in a concentration-dependent manner (*p* < 0.001), compared with no-punicalagin control (Figure 2B), suggesting that punicalagin treatment caused a significant fall in the intracellular pH of *V. parahaemolyticus*. Meanwhile, the membrane potential was determined with DiBAC_4_(3), a negatively charged anion slow-response probe that only emits fluorescence when it enters the cell and binds to proteins in the cytoplasm. An obvious increase in fluorescence occurred in the treated cells (*p* < 0.001) (Figure 2C), indicating cell membrane depolarization after punicalagin treatment.

### 3.3. Effects of Punicalagin on Membrane Permeability

**Figure 3:**
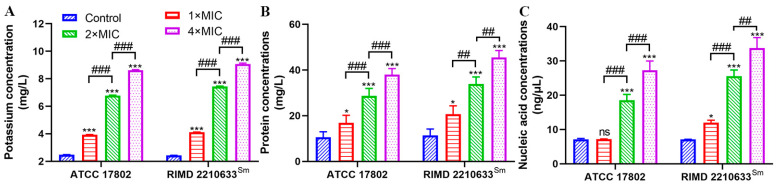
The extracellular concentration of potassium ions (**A**), proteins (**B**), and nucleic acids (**C**) of *V. parahaemolyticus* ATCC 17802 and RIMD 2210633^Sm^ treated with punicalagin. * *p* < 0.05, *** *p* ≤ 0.001 versus the control; ^##^ *p* ≤ 0.01, ^###^ *p* ≤ 0.001 for comparison between punicalagin treatments. ns, no significant difference.

K^+^ is the major cytoplasmic cation necessary for bacterial growth to assume several key functions, such as the activation of cytoplasmic enzymes, the maintenance of turgor pressure, and possibly the regulation of the cytoplasmic pH [20]. Punicalagin treatment induced a remarkable efflux of potassium in a dose-dependent manner (*p* < 0.001) (Figure 3A). The potassium efflux of *V. parahaemolyticus* ATCC 17802 and RIMD 2210633^Sm^ in the control was 2.48 mg/L and 2.44 mg/L, respectively. After treatment with 1×, 2×, and 4× MIC punicalagin, the efflux of potassium, separately, reached 3.94, 6.78, and 8.62 mg/L and 4.11, 7.44, and 9.07 mg/L, suggesting an enhanced permeability of the cytoplasmic membrane. In addition to ion, punicalagin increased the permeability for biomacromolecules. The intracellular protein releases from *V. parahaemolyticus* ATCC 17802 and RIMD 2210633^Sm^ were varied over a concentration range of 16.2 to 37.3 mg/L and 20.0 to 44.8 mg/L following 1×, 2×, and 4× MIC punicalagin (*p* < 0.05), which increased by 75.8–329.1% and 100.9–371.6% compared to the no-punicalagin control, respectively (Figure 3B). Moreover, punicalagin prompted the leakage of nucleic acid (Figure 3C). Compared to the control, punicalagin at 2× and 4× MIC caused the extremely notable leakage of nucleic acid (a 159.1% and 280.7% increase) for *V. parahaemolyticus* ATCC 17802 (*p* < 0.001), although there were no significant differences at 1× MIC, while a more pronounced leakage of nucleic acid was demonstrated as 68.7, 259.2, and 374.2% elevation for RIMD 2210633^Sm^ treated with 1×, 2×, and 4× MIC punicalagin (*p* < 0.05). The significant loss of cytoplasmic constituents implied irreversible damage to the cytoplasmic membrane.

**Figure 4:**
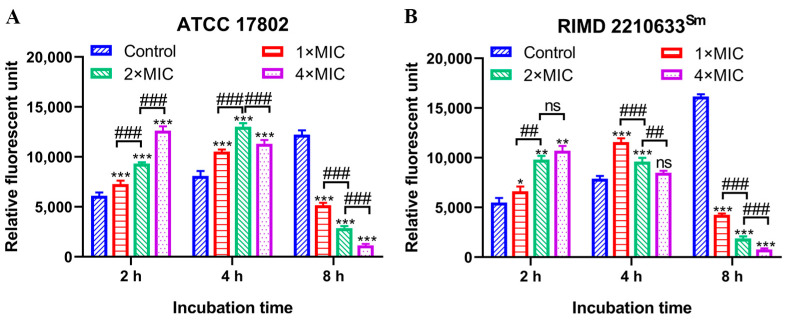
NPN uptake of *V. parahaemolyticus* ATCC 17802 (**A**) and RIMD 2210633^Sm^ (**B**) during punicalagin exposure. * *p* < 0.05, ** *p* ≤ 0.01, *** *p* ≤ 0.001 versus the control; ^##^ *p* ≤ 0.01, ^###^ *p* ≤ 0.001 for comparison between punicalagin treatments. ns, no significant difference.

NPN uptake assay demonstrated that punicalagin destabilized and weakened the outer membrane of *V. parahaemolyticus* cells (Figure 4). NPN is a hydrophobic fluorescent probe with low fluorescence absorption in an aqueous solution and enhanced fluorescence absorption in non-polar or hydrophobic environments. Once the outer membrane is damaged or the cell structure changed, NPN can enter the hydrophobic environment, resulting in brilliant fluorescence. When the bacterial cell membrane is severely damaged, NPN will escape from the hydrophobic environment, leading to a low fluorescence absorption value [21]. Compared to the control, punicalagin at 1×, 2×, and 4× MIC induced a profound NPN uptake for *V. parahaemolyticus* ATCC 17802 of 19.1%, 52.5%, and 106.6% amplification (Figure 4A), together with increases of 20.7%, 78.4%, and 95.0% for RIMD 2210633^Sm^ (Figure 4B) at 2 h. The NPN fluorescence intensity decreased with the prolongation of action time and the increased punicalagin concentrations. At 8 h, the fluorescence intensity of NPN in the treated cells was significantly lower than that at 2 h and 4 h, and it decayed observably in a dose-dependent manner compared with the no-punicalagin control (*p* < 0.001), which suggests that punicalagin treatment caused severe damage to the outer membrane and envelope structure.

### 3.4. Effects of Punicalagin on Membrane Integrity

**Figure 5:**
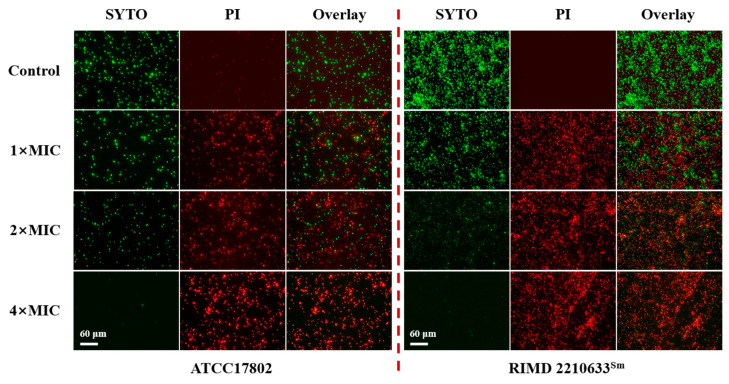
Fluorescence microscope images of *V. parahaemolyticus* ATCC 17802 and RIMD 2210633^Sm^ treated with or without punicalagin.

SYTO and PI are two fluorescent dyes widely used to detect cell membrane integrity. SYTO can freely penetrate into the cell membrane and bind to nucleic acid while emitting a green fluorescence. PI can only pass through the damaged cell membrane to bind with the nucleic acid and emit a red fluorescence. As shown in Figure 5, in the control, *V. parahaemolyticus* ATCC 17802 and RIMD 2210633^Sm^ all exhibited strong green fluorescence, indicating intact cell membrane. In contrast, an increasing red fluorescence with a declining green fluorescence occurred in treated cells with the increase in punicalagin treatment concentrations.

### 3.5. FE-SEM Observation

**Figure 6:**
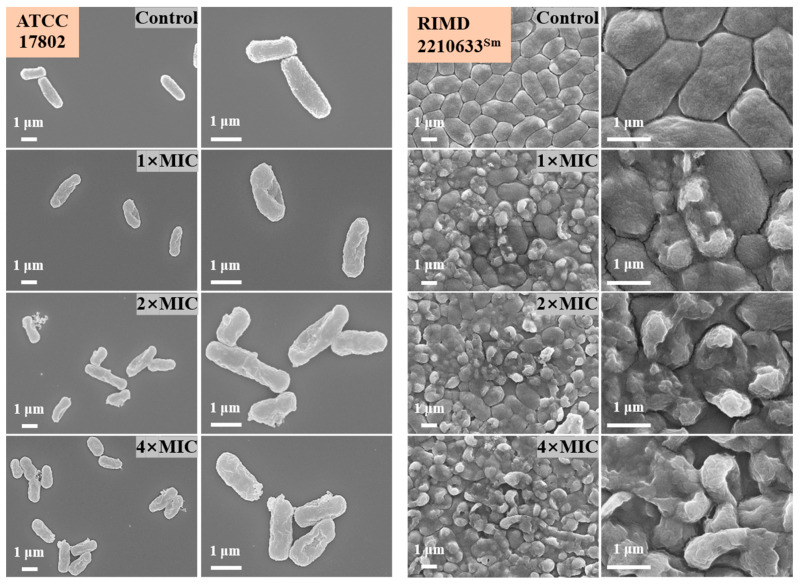
FE-SEM images of *V. parahaemolyticus* ATCC 17802 and RIMD 2210633^Sm^ exposed to punicalagin.

To visualize the effect of punicalagin on cell injury, FE-SEM was used to observe the morphology changes of *V. parahaemolyticus* after treatment with punicalagin. As shown in Figure 6, in the control, *V. parahaemolyticus* ATCC 17802 and RIMD 2210633^Sm^ all exhibited a typical pleomorphic structure of Gram-negative coccobacillus, showing full and plump rod-shapes or slightly curved arc shapes with an intact cell envelope as well as a regular and smooth surface. By comparison, visible shrinkage and wrinkles for *V. parahaemolyticus* ATCC 17802 and several more serious deformations, including collapse and cell lysis, for RIMD 2210633^Sm^ arose in punicalagin-treated cells at 1× MIC. What is more, cells treated with 2× and 4× MIC punicalagin displayed extensive cell deformations, cell membrane disruption, and the leakage of cytoplasm, with the severity of cell damage increasing in a dose-dependent manner.

### 3.6. Interaction of Punicalagin with Cell Biomolecules

**Figure 7:**
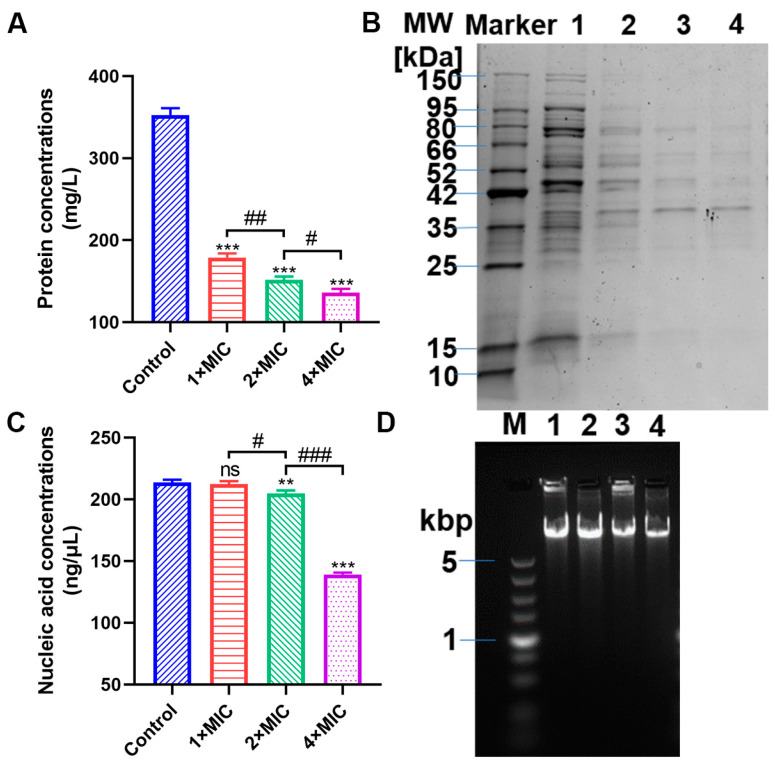
The protein concentration (**A**) and SDS-PAGE profile (**B**) of the bacterial protein of *V. parahaemolyticus* ATCC 17802 following punicalagin treatment. Lane 1: control; Lanes 2, 3, and 4: sample treated with 1×, 2×, and 4× MIC punicalagin, respectively. The DNA concentration (**C**) and AGE pattern of genomic DNA (**D**) of *V. parahaemolyticus* ATCC 17802. Lane M: marker; Lane 1: control; Lanes 2, 3, and 4: sample treated with 1×, 2×, and 4× MIC punicalagin, respectively. ** *p* ≤ 0.01, *** *p* ≤ 0.001 versus the control; ^#^ *p* < 0.05, ^##^ *p* ≤ 0.01, ^###^ *p* ≤ 0.001 for comparison between punicalagin treatments. ns, no significant difference.

The damaged cell membrane and injured cells permitted punicalagin to reach the inner structure of the cell, spurring us to investigate in depth the interaction of punicalagin with intercellular targets like the pillar components of bacteria (protein and DNA), which might play a part in the overall antimicrobial activity. As exhibited in Figure 7, the protein concentrations of *V. parahaemolyticus* ATCC 17802 in the control were 352.6 mg/L, and they decreased by 49.3, 56.9%, and 61.4% (*p* < 0.001) after treatment with punicalagin in a dose-dependent manner (Figure 7A). Correspondingly, SDS-PAGE images visually indicated the proteins with molecular weights ranging from 10 to 150 kDa and strong intensities for the control. However, the protein band intensities displayed an obvious gradual weakening except for the protein of ~38 kDa, with enhanced intensities with the increase in punicalagin concentration (Figure 7B). Likewise, the DNA concentration of *V. parahaemolyticus* treated with punicalagin was markedly reduced at 2× and 4× MIC (*p* < 0.01), while no significant decline occurred in cells treated with 1× MIC punicalagin (*p* > 0.05) compared to the control (Figure 7C). Here, AGE images of genomic DNA extracted from the treated cells demonstrated the decreased fluorescent intensity; nevertheless, the immigration rate of these bands was always consistent with that of the control (Figure 7D), implying the leakage of genomic DNA and very trivial or no interaction between punicalagin and genomic DNA.

**Figure 8:**
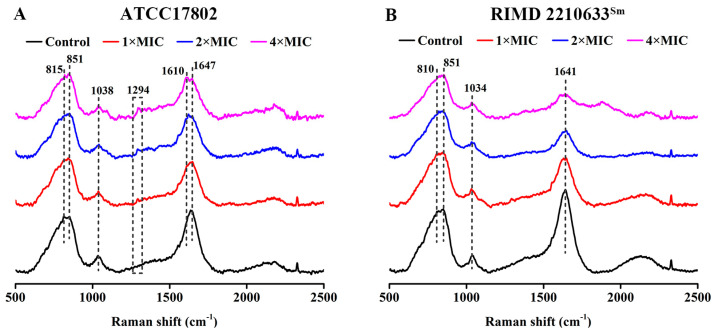
Raman spectrum of *V. parahaemolyticus* ATCC 17802 (**A**) and RIMD 2210633^Sm^ (**B**) exposed to punicalagin.

Further, we also investigated the possible effect of punicalagin on biochemical compositions of *V. parahaemolyticus* cells via laser Raman spectroscopy. The two *V. parahaemolyticus* strains showed very similar spectral features, with the most intense peaks near 810, 851, and 1641 cm^−1^ and a moderate peak around 1036 cm^−1^, whether treated with punicalagin or not (Figure 8), which most likely due to the vibrational modes of biomolecules on the outer membrane of *V. parahaemolyticus*. Raman peaks assignments were summarized in Table 2. Compared to the control, the Raman spectrum of *V. parahaemolyticus* ATCC 17802 treated with punicalagin incurred a slightly decreased intensity of the spectral peaks at 815 and 851 cm^−1^ and a visible reduction for RIMD 2210633^Sm^ (Figure 8). The peak around 815 cm^−1^ was mainly related to the C–O–P–O–C–RNA binding of nucleic acids and tyrosine, while the peak at 851 cm^−1^ was also connected with C–C proline stretching and C–O–C stretching, as well as tyrosine, which were assigned to proteins and saccharides. In addition, the peak around 1036 cm^−1^, likely attributed to the C–H in-plane deformation of phenylalanine (proteins), as well as the C–O and C–C stretching of saccharides and the C–N stretching of nucleic acids, was almost unaffected by the addition of punicalagin (Figure 8). On the other hand, a new peak appeared near 1294 cm^−1^, mostly in connection with the CH_2_ deformation of lipids, though also possibly with amide III or cytosine in punicalagin-treated cells of *V. parahaemolyticus* ATCC 17802 (Figure 8A). Moreover, the peak around 1647 cm^−1^, primarily involving the amide I region of proteins (and perhaps the C=O stretching of lipids), presented an obviously reduced intensity for the two *V. parahaemolyticus*, and especially the RIMD 2210633^Sm^ strain after punicalagin treatment (Figure 8).

## 4. Discussion

*V. parahaemolyticus* is the leading cause of global seafood outbreaks. Although the mechanism by which *V. parahaemolyticus* causes infection has yet to be clearly demonstrated, the presence of tdh and/or trh genes has been recognized as a major pathogenic risk [27,28]. More than that, tlh gene (usually as a species marker for detection of *V. parahaemolyticus*) was also found to be pathogenic in the presence of lecithin, which exists in most living organisms [29]. Worse, the majority of *V. parahaemolyticus* isolates from seawater samples and aquatic products have exhibited multidrug resistance (mainly to ampicillin and streptomycin) [4,5,30,31]. However, our previous [13] and current results demonstrate the strong bacteriostatic and bactericidal effects of punicalagin against different serotypes and genotypes of *V. parahaemolyticus*, including the RIMD 2210633^Sm^ strain. The MIC and MBC values of punicalagin were 150–200 μg/mL and 200–300 μg/mL, respectively (Table 1). These values are much lower than the MIC (781.25–3125 μg/mL) and MBC (1562.5–6250 μg/mL) values of detergents (as antibacterial agents separately used in the food industry, the household, and for cleaning purposes) against *V. parahaemolyticus* ATCC 17802 [32]. Taguri et al. previously determined the MIC of 10 different plant polyphenols against *V. parahaemolyticus* ATCC17802. Their results indicated that the MIC value of punicalagin was on a par with that of epigallocatechin, EGCG, and tannic acid, castalagin, and lower than that of prodelphinidins, geraniin, theaflavins, and loquat procyanidins [11]. Meanwhile, a bacterial growth assay verified the bactericidal activity of punicalagin against *V. parahaemolyticus*, with the significant decrease in bacterial counts being dependent on the concentration and the time of exposure (Figure 1). Similar results were observed in *Staphylococcus aureus* and *Salmonella typhimurium* [15,33]. These results, along with ours, raised the prospects of punicalagin in developing an effective antimicrobial.

It has been reported that phenolic compounds usually act by interfering with the basic membrane functions [8]. In our study, the FE-SEM observation suggested that the envelope structure of *V. parahaemolyticus* was severely damaged by the addition of punicalagin. At 1× MIC, it principally showed surface shrinkage. Cell collapse and lysis occurred more at 2× and 4× MIC (Figure 6). Consistent with this observation, the envelope permeability and several transporting processes across the cytoplasmic membrane were also varied. These may be the primary targets of punicalagin exerting bactericidal actions. In this section, we will discuss these variations in detail.

It is known that Gram-negative bacteria are naturally hard to kill because of their complex cell envelope that consists of an outer membrane and an inner membrane, with the peptidoglycan cell wall and periplasmic space in between. In the current study, the uptake of NPN indicated that punicalagin impaired the outer membrane permeability of *V. parahaemolyticus*, and its effect depends upon the concentration and the time of exposure (Figure 4). According to Rojas et al. [34], the outer membrane is an important mechanical element in Gram-negative bacteria and has clear consequences for antibacterial therapy. Compromising the outer membrane, chemically or genetically, greatly increased the deformation of the cell envelope and induced elevated levels of cell lysis upon mechanical perturbation and L-form proliferation [34]. Several plant phenolics such as thymol, carvacrol, resveratrol, and pinosylvin have earlier been reported to disintegrate the outer membrane of different bacteria [35,36,37], which may be one of the antibacterial mechanisms of punicalagin.

Our results also showed that exposure to punicalagin led to a reduction in the intracellular ATP level (Figure 2A). ATP is necessary for the survival and metabolism of living organisms, and any alteration or interruption of cellular bioenergetics may be another vital way of triggering cell death. Intracellular ATP reduction may arise from (1) unabated hydrolysis of ATP by the proton-pumping ATPase; (2) blocked ATP synthesis by the inhibition of energy material uptake pathways or disrupting the proton motive force; and (3) increased membrane permeability leading to leakage of internal ATP [20,38,39,40]. We assumed that ATP depletion in *V. parahaemolyticus* resulted from the leakage through the compromised membrane since we observed the damaged envelope structure by punicalagin, whereas we also observed that punicalagin caused a decrease in intracellular pH and membrane potential (Figure 2B,C). The results possibly imply the dissipation of the proton motive force necessary for ATP synthesis. On the other hand, Chen and Montville [41] suggested that loss of ATP was due to an accelerated hydrolysis from attempts by the cell to maintain proton motive force. Kang et al. [42] recently reported that ATP depletion of *Shigella flexneri* induced by ferulic acid might be due to cell membrane damage promoting the loss of internal ATP and affecting intracellular ATP synthesis as well as ATPase activity. According to a recent study, punicalagin can cause complete inhibition of *E. coli* ATP synthase [43], which may partially contribute to the antibacterial properties. Either way, it is concluded that the energy-transducing processes and cytomembrane homeostasis of *V. parahaemolyticus* were severely disturbed on exposure to punicalagin. And the release of cytoplasmic ions and molecules from *V. parahaemolyticus* cells treated with punicalagin amounted to further direct evidence for the permeability perturbation of the cytoplasmic membrane (Figure 3). Therefore, the uptake of membrane-impermeant probe PI increased in punicalagin-treated cells (Figure 5). Correspondingly, the concentrations of proteins and nucleic acids in *V. parahaemolyticus* decreased after punicalagin treatment (Figure 7A,C). In accordance with these results, Xu et al. [33] previously reported that punicalagin induced membrane damage and increased the permeability of the cytoplasmic membrane in *S. aureus*, with an immediate and accelerated K^+^ efflux at 2× MIC. Ashrafudoulla et al. [44] reported that eugenol destroyed the membrane integrity of *V. parahaemolyticus* and enhanced the leakage of intracellular nucleic acids and proteins in a dose- and time-dependent manner.

Moreover, punicalagin could interact with the protein of *V. parahaemolyticus* (Figure 7B). In contrast to the present study, Chen et al. [45] reported that curcumin (0.5–5 μM) did not possess negative effects on the protein integrity of *V. parahaemolyticus*. It is likely that different phenolic compounds may act according to various possible modes of antibacterial action due to their diverse chemical structures, antimicrobial sensitivity, and the various molecular mechanisms of antimicrobial activity. Next, the integrity of genomic DNA was largely unaffected by the addition of punicalagin (Figure 7D). Similarly, Chen et al. [45] reported that no significant change in the genomic DNA was observed in *V. parahaemolyticus* after curcumin treatment, implying that there was no obvious toxicity against *V. parahaemolyticus*. Further, Raman spectroscopic analysis showed a decrease in C–O–P–O–C–RNA binding, tyrosine, C–C proline stretching, carbohydrate C–O–C stretching, and especially the amide I region of proteins, together with the emergence of CH_2_ deformation for *V. parahaemolyticus* ATCC17802 (Figure 8A), indicating that punicalagin disrupted the outer membrane components (mainly the protein) and the bacterial cells. And the decrease was more obvious in *V. parahaemolyticus* RIMD 2210633^Sm^ (Figure 8B), suggesting more extensive envelope disruption and cell damage. These results further supported that punicalagin treatment induced the destruction of the biochemical compositions (mainly the protein) and envelope structures of *V. parahaemolyticus* cells.

To summarize, punicalagin attacked multiple targets of *V. parahaemolyticus* and could affect the envelope integrity, energy-transducing processes, and protein expression of *V. parahaemolyticus* cells. Targeting the cell envelope helps reverse antimicrobial resistance (AMR) in Gram-negative bacteria as the Gram-negative cell envelope is home to many different AMR determinants [46,47]. Moreover, compounds that exert an antimicrobial effect through a multi-target mechanism may also help improve the antibacterial efficacy and avoid AMR. A previously published study characterized a compound that killed both Gram-negative and Gram-positive organisms through a dual-targeting mechanism of action (folate metabolism and bacterial membrane integrity) with undetectably low resistance frequencies [48].

Future research could be conducted on characterizing the individual properties of food matrices, testing the synergistic effect of combined control measures to optimize the innate antimicrobial nature of punicalagin, and this could also help in developing potent measures against *V. parahaemolyticus*.

## 5. Conclusions

This study demonstrated that punicalagin exhibited an effective antibacterial effect against *V. parahaemolyticus*, revealing that one of the antibacterial mechanisms of punicalagin was its targeting of the cell envelope. Punicalagin destroyed the cell morphology and structure, compromised the permeability and integrity of the outer membrane and inner membrane, triggered the leakage of cytoplasmic constituents, and extinguished the electrochemical proton gradient, causing membrane depolarization and a decrease in intracellular pH, thereby resulting in the depletion of internal ATP and eventually cell lysis and death. Moreover, punicalagin could inhibit *V. parahaemolyticus* growth by interfering with the bacterial proteins. Punicalagin could be utilized as a natural antibacterial agent for the control of *V. parahaemolyticus* in food systems.

## Figures and Tables

**Table 1:** Minimum inhibitory concentrations (MICs) and minimum bactericidal concentration (MBC) of punicalagin against different strains of *V. parahaemolyticus*.

Strain	Serotype	Genotype	Origin	MIC (μg/mL)	MBC (μg/mL)
ATCC 17802	O1	tdh−/trh+/tlh+	Shirasu food poisoning	200	200
ATCC 33847	O4	tdh+/trh−/tlh+	Gastroenteritis	150	200
RIMD 2210633^Sm^	O3:K6	tdh+/trh−/tlh+	Clinical isolation	200	300

**Table 2:** Assignments of Raman peaks of *V. parahaemolyticus* exposed to punicalagin.

Raman Shift (cm^−1^)	Assignment	Macromolecular Assignment	Reference
810–820	C–O–P–O–C–RNA binding; tyrosine	Nucleic acids; proteins	[22,23]
851	Buried tyrosine; C–C proline stretching; C–O–C stretching	Proteins; saccharides	[22,23]
1031–1046	C–H in-plane deformation-phenylalanine/proline; C–O–C stretching; C–N stretching	Proteins; saccharides; nucleic acids	[22,23]
1295	CH_2_ deformation; amide III; cytosine	Lipids; proteins; nucleic acids	[22,23,24]
1641–1650	Amide I; unsaturated lipids	Proteins; lipids	[25,26]

## Data Availability

The original contributions presented in the study are included in the article. Further inquiries can be directed to the corresponding author.
